# Psychosocial support for adolescents and youth living with HIV during COVID-19: A differentiated approach is needed

**DOI:** 10.4102/sajhivmed.v23i1.1379

**Published:** 2022-04-14

**Authors:** Carol L. Tait, Njabulo Mbanda, Rudairo Tumba, Marnie J. Vujovic, Kate Rees

**Affiliations:** 1 Anova Health Institute, Johannesburg, South Africa; 2 School of Public Health, Faculty of Medicine, University of the Witwatersrand, Johannesburg, South Africa

South Africa has a high number of adolescents and youth accessing HIV treatment. Psychosocial support is needed to support retention and suppression in this group, who are navigating this life stage while living with HIV. In Johannesburg, this was traditionally delivered through facility-based Youth Care Clubs (YCC), which were adapted to virtual delivery due to coronavirus disease 2019 (COVID-19). Despite the benefits, several challenges were identified during implementation. We aimed to improve the virtual support offering through utilising an alternative reverse-billed platform.

Existing and alternative platforms were reviewed with collaborating partners. Challenges of WhatsApp-based YCC included data costs, access to devices, lack of interaction and inability to see or hear group members. A reverse-billed platform was identified as a possible solution, requiring no data or applications. Staff were trained and existing virtual groups selected based on age and current activity. Data are presented for June 2021 to August 2021.

Of the 91 existing virtual groups, 31 were selected for the new platform. Four days/month were selected to minimise running costs, based on participant preferences. Several trainings were needed to cover all facilitators. A step-by-step guide was introduced to mitigate technical challenges. Ten tablets were procured to assist participants without devices. On average 255 participants planned to join groups each month. Of these, 110 joined (43%). Benefits included live discussion, video sharing, and feeling more connected due to voice and video sharing. Groups also continued discussion on WhatsApp platforms. Challenges included network coverage (mainly due to electricity cuts), difficulty navigating the platform, lack of flexibility over group times, and participants not joining as planned.

The new platform offered additional benefits; however, despite data-free access, network connectivity and other challenges affected participation. A differentiated approach continues to be needed, with more flexibility, to enable access to retention support for adolescents and youth.

The authors would like to thank the implementing psychosocial team from Anova Health Institute for embracing new ways to care for adolescents and youth despite the challenges, and also to the Department of Health teams for managing these clients at health facilities with dedication. This work would not be possible without the generous support and donation of our funders.

**Ethical considerations:** Approval to conduct the study was received from the Human Sciences Research Council Ethics Committee (REC3/22/08/18).

**Funding information:** A COVID-relief grant was received through an existing funder to implement virtual support for adolescents. The Anova Health Institute non-profit company is supported by the United States President’s Emergency Plan (PEPFAR) programme via the United States Agency for International Development (USAID) under the Cooperative Agreement number AID-674-A-12-00015.

